# Characteristic of Uterine Rhabdomyosarcoma by Algorithm of Potential Biomarkers for Uterine Mesenchymal Tumor

**DOI:** 10.3390/curroncol29040190

**Published:** 2022-03-28

**Authors:** Saya Tamura, Takuma Hayashi, Tomoyuki Ichimura, Nobuo Yaegashi, Kaoru Abiko, Ikuo Konishi

**Affiliations:** 1 National Hospital Organization Kyoto Medical Center, Department of Obstetrics and Gynecology, Kyoto 612-8555, Japan; nho.kmc.rc@hotmail.com (S.T.); hiroyukiaburatani@yahoo.co.jp (K.A.); ikuokonishi08@yahoo.co.jp (I.K.); 2 Section of Cancer Medicine, National Hospital Organization Kyoto Medical Center, Kyoto 612-8555, Japan; 3 START-Program, Japan Science and Technology Agency (JST), Tokyo 102-8666, Japan; 4 Department of Obstetrics and Gynecology, Osaka City University School of Medicine, Osaka 545-8586, Japan; kenjisano12@yahoo.co.jp; 5 Department of Obstetrics and Gynecology, Tohoku University School of Medicine, Miyagi 980-8575, Japan; nobuoyaegashi@yahoo.co.jp; 6 Department of Obstetrics and Gynecology, Kyoto University School of Medicine, Kyoto 606-8501, Japan

**Keywords:** rhabdomyosarcoma, leiomyoma, leiomyosarcoma, mesenchymal tumor

## Abstract

Background/Aim: Patients with uterine sarcoma comprise 2–5% of all patients with uterine malignancies; however, the morbidity of uterine sarcoma is low compared with that of other gynecological cancers. For many cases, malignant uterine tumors are diagnosed during follow-up of benign uterine leiomyoma. Of the uterine sarcomas, rhabdomyosarcoma is considered a mixed tumor containing components of epithelial cells and mesenchymal cells. Therefore, the onset of primary uterine rhabdomyosarcoma during follow-up of uterine leiomyoma is extremely rare. Rhabdomyosarcoma is a relatively common malignant tumor in children, but rhabdomyosarcoma in adults is extremely rare, accounting for approximately 3% of all patients with soft tissue sarcoma. Rhabdomyosarcoma in children is highly sensitive to chemotherapy and radiation therapy; however, the response to chemotherapy and radiation therapy in adult rhabdomyosarcoma is low and survival in adult rhabdomyosarcoma with metastatic lesions to other organs is approximately 14 months. We experienced a case of pleomorphic rhabdomyosarcoma during the follow-up of a uterine leiomyoma. Materials and Methods: We examined the oncological properties of uterine rhabdomyosarcoma in adults using molecular pathological techniques on tissue excised from patients with uterine leiomyoma. Result: A differential diagnosis was made for this case by molecular pathology, which included candidate biomarkers for uterine smooth muscle tumors. The oncological nature of uterine rhabdomyosarcoma was found to be similar to the oncological properties of uterine leiomyosarcoma. However, in uterine rhabdomyosarcoma, LMP2/β1i-positive cells were clearly observed. Conclusion: It is expected that establishing a diagnostic and treatment method targeting characteristics of mesenchymal tumor cells will lead to the treatment of malignant tumors with a low risk of recurrence and metastasis.

## 1. Introduction

Uterine mesenchymal tumors are broadly classified into two types: benign and malignant tumors. However, for uterine mesenchymal tumors, cells with various histological types and cell morphologies are mixed. Furthermore, the components contained within the cells also vary. Therefore, surgical pathological diagnosis for uterine mesenchymal tumor is challenging. Uterine sarcoma in gynecologic oncology is a rare disease and, in most cases, it develops in the uterine body. Therefore, sarcomas that occur outside of the uterus, such as the vagina, vulva, and ovaries, are extremely rare. In 2020, the International Classification for Gynecologic Tumors published by the World Health Organization classified uterine sarcoma as cancer of the uterine body [1]. As a result, the histological types of uterine sarcoma are classified as uterine leiomyosarcoma, endometrial stromal sarcoma (low and high grade), and undifferentiated uterine sarcoma [2]. As a somewhat rare uterine sarcoma, the development of uterine adenosarcoma, which is a malignant tumor, may occur.

In clinical practice, histopathologically poor prognostic factors for uterine adenosarcoma include lymphovascular invasion, differentiation into uterine rhabdomyosarcoma, and overgrowth of sarcoma components [3]. Uterine adenosarcoma is a mixed tumor consisting of components of benign glandular epithelial tissue and sarcoma tissue. Uterine adenosarcoma is known to form foliate polyp-like elevated lesions. The incidence of uterine adenosarcoma is only 1/9 of that observed for uterine carcinosarcoma [4]. The age of onset of uterine adenosarcoma is younger compared with that of uterine carcinosarcoma and the results of a clinical study with 100 patients indicated that the distribution by age was 14–89 years, with a median of 58 years [5]. The site of onset of uterine adenosarcoma is the uterine endometrium in 76% of all cases, the cervix endometrium in 6%, and the myometrium layer of the uterus in 4%. Atypical components of the sarcomatous tissue of uterine adenosarcoma are not always pathologically evident. Uterine adenosarcoma is diagnosed as an intimal or cervical polyp and repeated recurrences occur, thus regular examination is required. As a treatment for uterine adenosarcoma, like other uterine sarcomas, surgery by simple abdominal hysterectomy and bilateral adnexectomy is standard practice. However, as a clinical treatment for uterine adenosarcoma, the effectiveness of lymph node dissection and post-operative treatment has not been established. Compared with other uterine sarcomas, uterine adenosarcoma exhibits a better prognosis, and clinical studies have indicated that the five-year survival rate is 79% in pre-operative stage I cases and 48% in cases considered stage III [4].

The pathological findings of uterine adenosarcoma are characterized by spindle-shaped cells displaying significant fission. In many cases of uterine adenosarcoma, the expression of cluster of differentiation (CD) 10, an epithelial cell marker, is diffusely positive, and the expression of alpha smooth muscle actin (αSMA) and desmin, a marker of smooth muscle cells, is negative [6,7]. In addition, in cases of uterine adenosarcoma, the expression of the hormone receptors, estrogen receptor (ER) and progesterone receptor (PgR), may be positive or negative [8,9]. Thus, the expression of these receptors differs depending on the case. Surgical pathology of uterine rhabdomyosarcoma is performed based on the differentiation of uterine adenosarcoma into uterine rhabdomyosarcoma and the molecular pathological characteristics of uterine rhabdomyosarcoma. For uterine rhabdomyosarcoma, immunohistochemical expression of desmin, muscle-specific actin, myogenin, myogenic and differentiation antigen 1 (MyoD1) is observed, and the expression status of these markers can be used as a diagnostic reference for uterine rhabdomyosarcoma [10].

Using surgical pathology, we examined neonatal-sized uterine tumors, pelvic lymph nodes, para-aortic lymph nodes, and left supraclavicular lymph nodes with bulky mass. The onset of uterine leiomyosarcoma and malignant lymphoma was suspected based on a significantly raised abdomen, a large mass on the left subclavian lymph node, ultrasonographic images, computed tomography images, and high lactic acid dehydrogenase (LDH) levels (3631 U/L). However, based on the results, the patient’s uterine tumor was diagnosed as a rhabdomyosarcoma originating from the uterus and metastasis to the left supraclavicular lymph node. The frequency of rhabdomyosarcoma at different sites within the female genital tract varies by histological subtype [11]. Pleomorphic rhabdomyosarcoma typically occurs in the corpus. In the lower female genital tract, embryonal rhabdomyosarcoma is most common in the vagina in children and in the cervix and corpus in adolescents and adults, whereas alveolar rhabdomyosarcoma is most common in the vulva. We examined the difference between uterine leiomyosarcoma and uterine rhabdomyosarcoma using molecular pathological techniques. Our results provide a new molecular marker for the diagnosis of gynecologic tumors.

## 2. Materials and Methods

### 2.1. Tissue Collection

A total of 101 patients between 32 and 83 years of age and diagnosed as having smooth muscle tumors of the uterus were selected from pathological files. Serial sections were cut from at least two tissue blocks from each patient for hematoxylin and eosin staining and immunostaining. All tissues were used with the approval of the Ethical Committee of Shinshu University after obtaining written consent from each patient. The pathological diagnosis of uterine smooth muscle tumors was performed using established criteria (Hendrickson and Kempson, 1995) with some modification. Briefly, usual leiomyoma (usual LMA) was defined as a tumor showing typical histological features with a mitotic index (MI) (obtained by counting the total number of mitotic figures (MFs) in 10 high-power fields (HPFs)) of <5 MFs per 10 HPFs. Cellular leiomyoma (cellular LMA) was defined as a tumor with significantly increased cellularity (>2000 myoma cells/HPF) and a MI < 5, but without cytologic atypia. Bizarre leiomyoma (BL) was defined as a tumor either with diffuse nuclear atypia and a MI < 2 or with focal nuclear atypia and a MI < 5 without coagulative tumor cell necrosis. A tumor of uncertain malignant potential (UMP) was defined as tumor with no mild atypia and a MI < 10 but with coagulative tumor cell necrosis. Leiomyosarcoma (LMS) was diagnosed in the presence of a MI > 10 with either diffuse cytologic atypia, coagulative tumor cell necrosis, or both. Of the 105 smooth muscle tumors, 52 were diagnosed as LMA, 3 were BL, 2 were intravenous leiomyomatosis, 58 were uterine LMS, 1 was uterine LANT-like tumor, and 2 were uterine rhabdomyosarcoma. Of the 58 LMS, 48 were histologically of the spindle-cell type and 10 were of the epithelioid type. The clinical stage of the LMS patients was stage I in 11 cases, stage II or III in 31 cases, and stage IV in 16 cases. Protein expression studies with cervix epithelium and carcinoma tissues were performed using tissue array (uterus cancer tissues, AccuMax Array, Seoul, Korea). Details about tissue sections are indicated in the manufacturer’s information (AccuMax Array).

### 2.2. Immunohistochemistry (IHC)

IHC staining for caveolin-1, cyclin B, cyclin E1, large multifunctional peptidase 2/β1i (LMP2/β1i), Ki-67, desmin, and myogenin was performed using serial human uterine mesenchymal tumor sections obtained from patients with uterine mesenchymal tumor. A monoclonal antibody against yclin E1 (CCNE1/2460) was purchased from Abcam (Cambridge Biomedical Campus, Cambridge, UK) and a monoclonal antibody against Ki-67 (clone MIB-1) was purchased from Dako Denmark A/S (DK-2600 Glostrup, Denmark). Monoclonal antibodies against desmin (clone RM234) and myogenin (clone MGN185) were purchased from Gene Tex, Inc. (Irvine, CA, USA). The monoclonal antibody against caveolin-1, cyclin B1, LMP2/β1i were purchased from Santa Cruz Biotechnology Inc. (Santa Cruz, CA, USA). IHC was performed using the avidin–biotin complex method as described previously [12,13,14]. Briefly, one representative 5-mm tissue section was cut from a paraffin-embedded sample of a radical hysterectomy specimen from each patient with a uterine mesenchymal tumor.

The sections were incubated with a biotinylated secondary antibody (Dako, DK-2600 Glostrup, Denmark) followed by the streptavidin complex (Dako). The completed reaction was developed using 3,39-diaminobenzidine and the slides were counterstained with hematoxylin. Normal myometrium portions in the specimens were used as positive controls. The negative controls consisted of tissue sections incubated with normal rabbit IgG instead of primary antibody. Shinshu University approved the experiments according to internal guidelines (approval no. M192). The expression of cyclin E and Ki-67 was indicated by brown 3,3′-diaminobenzidine, tetrahydrochloride (DAB) staining. Normal rabbit or mouse antiserum was used as a negative control for the primary antibody. The entire brown 3,3′-diaminobenzidine, tetrahydrochloride-stained tissue was scanned with a BZ-X800 digital microscope (Keyence, Osaka, Japan). Black dots indicated the expression of cyclin E and Ki-67.

IHC staining for CD31 and lymphatic vessel endothelial hyaluronan receptor 1 (LYVE-1) was performed on sections from the excised tissue. Briefly, tumor tissue sections were incubated with the appropriate primary antibodies at 4 °C overnight. Rabbit polyclonal antibodies to LYVE-1 (1:200) and a mouse monoclonal antibody to CD31 (1:200) were the primary antibodies. A monoclonal antibody for CD31 (clone JC/70A) was purchased from Gene Tex, Inc. (Irvine, CA, USA). The antibody for LYVE-1 (bs-20353R) was purchased from Bioss Inc. (Boston, MA, USA). Following incubation with an Alexa Fluor^®^ 488-conjugated anti-mouse IgG or Alexa Fluor^®^ 546-conjugated anti-rabbit IgG secondary antibody (1:200; Invitrogen, Waltham, MA, USA), the sections were washed, cover-slipped with mounting medium and 40,6-diamidino-2-phenylindole (DAPI) (Vectashield; Vector Laboratories, Burlingame, CA, USA), and visualized by confocal microscopy (Leica TCS SP8, Wetzlar, Germany) according to the manufacturer’s instructions. Normal rabbit or mouse antiserum was used as a negative control for the primary antibody. The experiments with human tissues were conducted at the National Hospital Organization Kyoto Medical Center in accordance with institutional guidelines (approval no. NHO H31-02).

### 2.3. Ethical Approval and Consent to Participate

This study was reviewed and approved by the Central Ethics Review Board of the National Hospital Organization Headquarters in Japan (Tokyo, Japan) and Shinshu University (Nagano, Japan). Ethical approval was obtained on 17 August 2019, and the code was NHO H31-02. The authors attended educational lectures on medical ethics in 2020 and 2021, which were supervised by the Japanese government. The completion numbers for the authors are AP0000151756, AP0000151757, AP0000151769, and AP000351128. Consent to participate was required as this research was considered clinical research. Subjects signed an informed consent form when they were briefed on the clinical study and agreed with content of the research. The authors attended a seminar on the ethics of experimental research using small animals on 2 July 2020 and 20 July 2021. They became familiar with the importance and ethics of animal experiments (National Hospital Organization Kyoto Medical Center and Shinshu University School of Medicine). The code number for the ethical approval for experiments involving small animals was KMC R02-0702.

## 3. Results

Case 1. On 31 May 2021, a 58-year old woman arrived at our hospital with a markedly swollen abdomen and swollen left supraclavicular lymph nodes (Supplementary Material S1). Hematological examination before surgical treatment revealed a high LDH value of 3631 U/L and she excreted a blood clot during long-term follow-up of uterine leiomyoma delivery. Therefore, we considered a diagnosis of uterine leiomyosarcoma accompanied by uterine leiomyoma. We performed molecular pathological analysis with multiple index markers for various soft tissue tumors including candidate biomarkers for uterine leiomyosarcoma using the tissues removed during surgical treatment.

Macroscopic findings of the excised tissue are shown below. The resected uterine tumor was markedly hypertrophied, and the findings of the cut surface indicated that the uterine wall was replaced by a white solid phyllodes lesion with necrosis. A part of the uterine tumor exhibited a hard nodule and calcification that appeared to be uterine leiomyoma.

**Figure 1:**
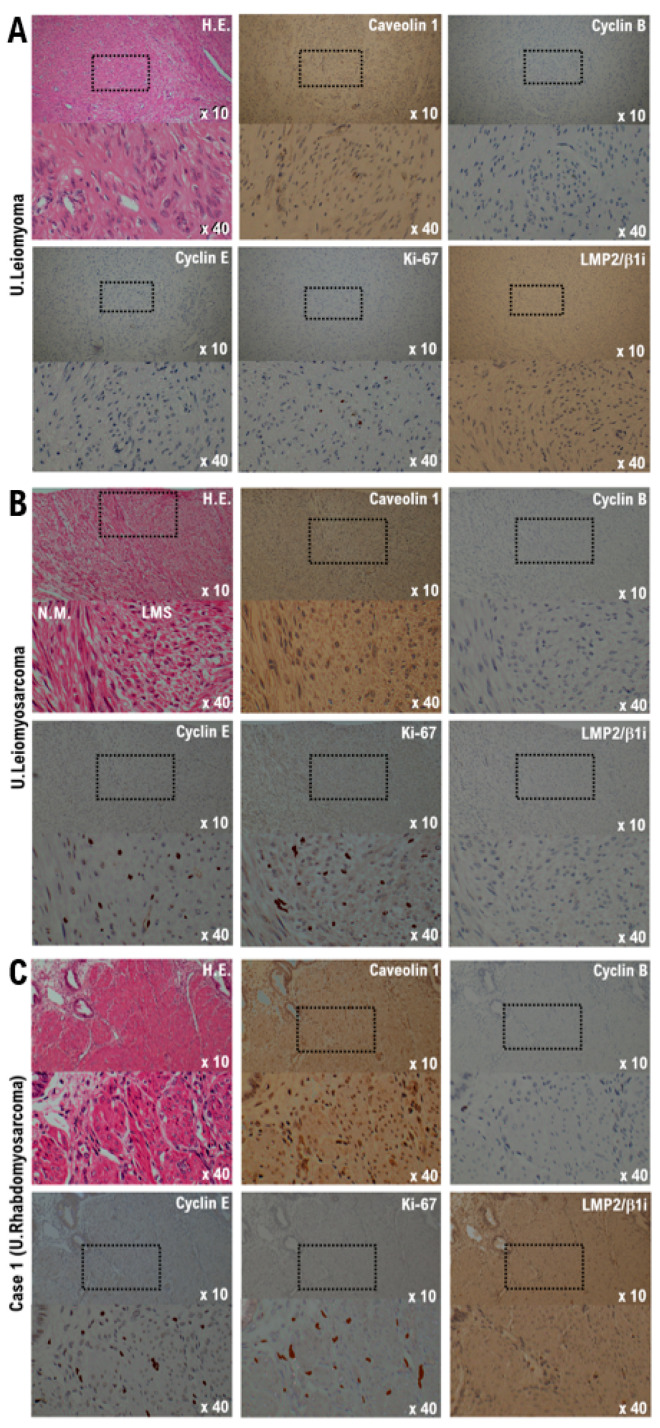
Differential expression of cyclin B, cyclin E, caveolin-1, ki-67, and LMP2/b1i as potential biomarkers in the normal myometrium, uterine leiomyoma, uterine leiomyosarcoma, and the Case 1 uterine tumor. (**A**) The image shows spindle cell leiomyoma. Low-power view (10× field) shows a well-circumscribed tumor nodule in the myometrium composed of broad fascicles of spindle cells. A high-power view (40× field) shows uterine leiomyoma (spindle cell) with bland cytological features, elongated nuclei, and fine nuclear chromatin. Immunohistochemistry of uterine leiomyoma tissue sections was performed using monoclonal antibodies. (**B**) The image shows uterine epithelioid leiomyosarcoma. Low power view (10× field) shows a uterine mass and an irregular interface with the myometrium composed of round to polygonal cells with granular eosinophilic cytoplasm. The presence of significant nuclear atypia and mitoses is evident. High-power view (40× field) shows tumor cells that are round to ovoid. The tumor cells have eosinophilic granular cytoplasm and irregular-shaped nuclei. Immunohistochemistry of the leiomyosarcoma tissue sections was performed using the appropriate monoclonal antibodies. (**C**) Case 1 uterine tumor appears as an admixture of round, polygonal, bizarre, or spindle cells, with marked atypia, with or without giant cells and rhabdomyoblasts. Some tumors invaded the lymphatic vessels. Low-power view (10× field) shows no obvious high-grade nuclear atypia or mitotic cell proliferation, and necrosis is observed. The high-power view (40× field) showing tumor cells with significant pleomorphism, whereas some are multinucleated and rhabdomyoblastic differentiation is evident. Immunohistochemistry with the of normal myometrium, leiomyoma, leiomyosarcoma, and the Case 1 uterine tumor was performed using appropriate monoclonal antibodies.

**Figure 2:**
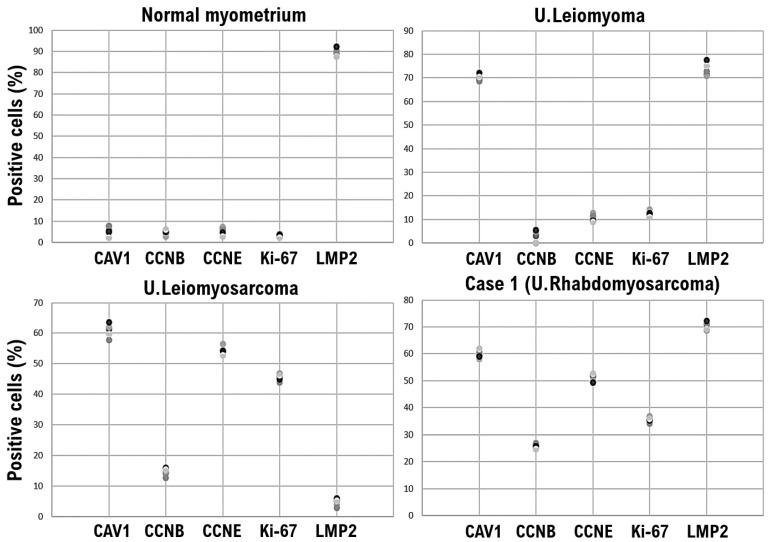
Significance of LMP2/b1i-positive uterine mesenchymal tumor cells in the Case 1 tumor as well as normal myometrium and uterine leiomyoma. Immunohistochemistry of normal myometrium, uterine leiomyoma, uterine leiomyosarcoma, and Case 1 uterine tumor tissues was performed using appropriate monoclonal antibodies. The tissues were randomly selected from normal myometrium, uterine leiomyoma, uterine leiomyosarcoma, and the Case 1 uterine tumor. Under a 40× field of view, the positive rates for five biomarkers were determined in four tissue sites under a microscope (Panthera Shimadzu Co. Ltd., Kyoto, Japan). The positive rates at each site for each tissue are displayed in a scatter plot. CAV1, caveolin-1; CCNB, cyclin B; CCNE, cyclin E; LMP2, LMP2/β1i.

The pathological findings are shown below. Significant necrosis was observed at the tumor site on the uterus and a viable mass remained primarily at the margin and around the blood vessels. Many tumor cells found in a patient’s uterine tumor are round-shaped cells or short-spindle-shaped cells with a high nuclear-cytoplasmic ratio (N/C). Furthermore, rhabdoid/rhabdomyoblastic cells, epithelioid cells, cells with bizarre large nuclei, large multi-nucleated cells, and spindle-shaped cells comprised the uterine tumor. Many fission cells were also observed in the tumor. Five factors (caveolin, cyclin B, cyclin E, LMP2/β1i, Ki-67) were evaluated as markers to differentiate uterine leiomyosarcoma from other mesenchymal tumors [15,16] (Figure 1 and Figure 2).

The expression of caveolin, a candidate biomarker for uterine mesenchymal tumors, was confirmed in the uterine leiomyoma and uterine tumor of the patient (Figure 1 and Figure 2). Mild expression of cyclin B, which is considered a biomarker for malignant tumors, was confirmed in the uterine leiomyosarcoma and uterine tumors of the patient (Figure 1 and Figure 2). Strong expression of cyclin E and Ki-67, biomarker candidates for malignant mesenchymal tumors, was also observed in the uterine leiomyosarcoma and uterine tumors (Figure 1 and Figure 2). Previous studies have indicated that the spontaneous onset of uterine leiomyosarcoma is observed in LMP2/β1i-deficient mice, which is one of the subunits of the immunoproteasome [17,18]. In human uterine leiomyosarcoma, the expression of LMP2/β1i is significantly reduced [17,18,19]. However, similar to normal uterine smooth muscle tissue and uterine leiomyoma, strong expression of LMP2/β1i was observed in the uterine tumor (Figure 1 and Figure 2). These results indicate that the uterine tumor appears to be malignant, as the possibility of a uterine leiomyosarcoma is low (Table 1).

**Figure 3:**
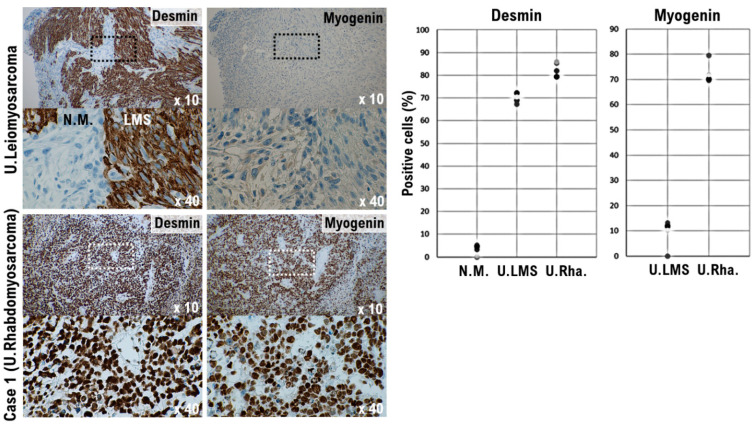
Significance of myogenin-positive uterine mesenchymal tumor cells in the Case 1 tumor. Differential expression of desmin and myogenin as potential biomarkers in normal myometrium, uterine leiomyosarcoma, and the Case 1 uterine tumor. The photograph shows uterine epithelioid leiomyosarcoma and normal myometrium. The low power view (10× field) shows the uterine mass irregular interface with normal myometrium, which is composed of round to polygonal cells with granular eosinophilic cytoplasm. The presence of significant nuclear atypia and mitoses is evident. A high-power view (40× field) shows tumor cells that are round to ovoid. The tumor cells have eosinophilic granular cytoplasm and irregular shaped nuclei. Immunohistochemistry (IHC) with the tissue sections of leiomyosarcoma was performed using the appropriate monoclonal antibodies (left upper panel). Case 1 uterine tumor is an admixture of round, polygonal, bizarre, or spindle cells, with marked atypia, with or without giant cells and rhabdomyoblasts. Some tumors invaded the lymphatic vessels. The low-power view (10× field) shows no obvious high grade nuclear atypia or mitotic cell proliferation. The high-power view (40× field) shows that tumor cells exhibit significant pleomorphism and some show multi-nucleated, rhabdomyoblastic differentiation. IHC with the Case 1 uterine tumor was performed using the appropriate monoclonal antibodies (left lower panel). The five tissue sites were randomly selected from normal myometrium, uterine leiomyosarcoma, and the Case 1 uterine tumor. In a 40× field of view, the positive rates for the two biomarkers were determined in three tissue sites under a microscope (Panthera Shimadzu Co. Ltd., Kyoto, Japan) (right panel). The positive rates at the sites for each tissue are shown in a scatter plot.

In histopathological diagnosis, desmin, myoglobin, myogenin, MyoD1, αSMA, and familial hyperinsulinemic hypoglycemia-35 (HHF-35) are used as markers for myogenic tissue. Desmin and HHF-35 are positive in both striated and smooth muscle tissues and are positive in many cases including uterine leiomyoma, rhabdomyosarcoma, uterine leiomyoma, and nodular fasciitis. Myogenin, MyoD1, and myoglobin are markers specific to striated muscle. Myogenin is highly sensitive and specific for rhabdomyosarcoma and is useful for differential diagnosis [24]. The results of immunohistochemical staining indicated strong expression of desmin, a molecular marker of muscle cells, in the uterine tumor and uterine leiomyosarcoma. Desmin expression was not evident in the normal uterine smooth muscle tissue (Figure 3). Strong expression of myogenin, a molecular marker of muscle cells, was observed in the uterine tumor, whereas high expression of myogenin was not observed in the uterine leiomyosarcoma (Figure 3). Given the positive status of myogenin, it was not considered to be a localized ectopic component of other tumors. Mild expression of synaptophysin, a neuroendocrine marker, and cytokeratin AE1/AE3, a marker of epithelial cells, was observed in the uterine tumor (Supplementary Material S2).

**Figure 4:**
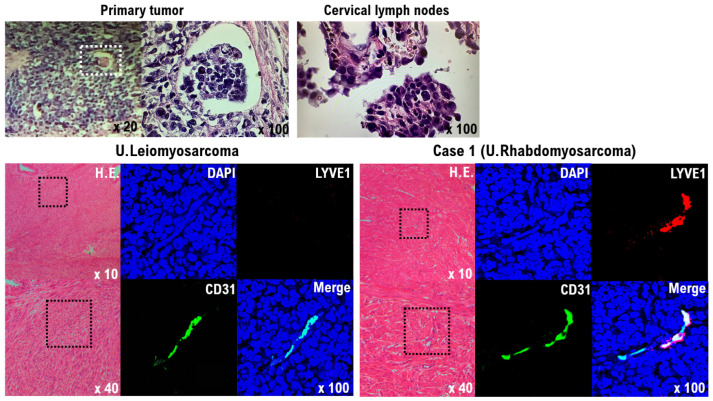
Significance of lymphatic endothelial cells in the primary Case 1 uterine tumor. Tumor cells infiltrating the lymph vessels within the uterine tumor are round cells with a high nuclear cytoplasmic ratio (N/C) (upper left panel). Circular cells with a high nuclear cytoplasm ratio (N/C) were also found in the biopsy from the tumor of the left supraclavicular lymph node (upper right panel). The results indicate that tumor cells from the primary uterine tumor formed metastases in the left supraclavicular lymph node by lymphatic metastasis. H&E staining and immunofluorescence of uterine leiomyosarcoma and the Case 1 uterine tumor with an anti-human CD31 (green) and anti-human LYVE-1 antibody (red) (lower panels). Human vascular endothelial cells and lymphatic endothelial cells were detected as CD31 and LYVE-1 double-positive cells. The tumor cells from the Case 1 uterine tumor appear to differentiate into lymphatic endothelial cell progenitors (lower light panel), but the tumor cells from the uterine leiomyosarcoma do not appear to have differentiated to lymphatic endothelial cell progenitors (lower left panel).

Circular cells with a high nuclear cytoplasm ratio (N/C) were found in the biopsy of the tumors of the left supraclavicular lymph node, as well as cells infiltrating the lymph vessels within the patient’s uterine tumor (Figure 4). In addition, intravascular infiltration by the uterine tumor cells was observed (Figure 4). Based on this observation, we determined that the tumor of the left supraclavicular lymph node was not a malignant lymphoma, but a lymph node metastasis derived from a malignant mesenchymal tumor originating in the uterus. Lymphatic endothelial cells (CD31 and LYVE1-positive cells), which were not found in the uterine leiomyosarcoma, were observed in the uterine tumor (Figure 4). Although lymphatic metastases are rarely found in uterine leiomyosarcoma, in pleomorphic rhabdomyosarcoma, distant metastases to other organs and lymph node metastases are common [25].

Based on these molecular pathological results, the patient was diagnosed with pleomorphic rhabdomyosarcoma. Pleomorphic rhabdomyosarcoma typically occurs in postmenopausal patients who present with abnormal vaginal bleeding [11]. The characteristics of pleomorphic rhabdomyosarcoma are consistent with the clinical findings.

Hematological examination before surgical treatment showed that the ovarian carcinoma antigen-125 (CA125) value was as high as 82, so it is possible that the giant uterine tumor may be a malignant tumor derived from epithelial cells, such as endometrial or ovarian cancer. No obvious malignant cells were observed in the fallopian tube tissue; however, endometriosis was observed in the tissues of the appendages, including the fallopian tubes. Thus, epithelial cells with nuclear swelling, so-called atypical endometriosis, were observed. Because CA125 is also produced in the peritoneum, thoracic membrane, or uterine/endometrial membrane, it is also elevated in benign and inflammatory diseases, such as benign ovarian cysts, endometriosis, uterine fibroids, inflammation, intestinal obstruction, pancreatitis, and cholecystitis. Therefore, the cause of high CA125 levels in patient serum is considered to be endometriosis.

## 4. Discussion

Rhabdomyosarcoma is the third most common solid childhood cancer outside of the central nervous system (after Wilms tumor and neuroblastoma). Rhabdomyosarcoma accounts for 3–4% of all childhood cancers. It belongs to a group of tumors known as soft tissue sarcoma and the number of patients with rhabdomyosarcoma is the highest among soft tissue sarcomas. The incidence of rhabdomyosarcoma in children is 4.3 out of 1 million annually. Two-thirds of the patients with rhabdomyosarcoma are under seven years of age. Rhabdomyosarcoma is more common in white ethnicities than in black ethnicities; in particular, it was shown that rhabdomyosarcoma occurs less frequently in black versus white girls. Additionally, the onset of rhabdomyosarcoma is slightly more common in boys than in girls. Multiple organ metastases occur in approximately 15–25% of children with rhabdomyosarcoma. The lung is the organ in which metastases most frequently occur, although metastases may also develop in the bone, bone marrow, and various lymph nodes. Uterine rhabdomyosarcoma consists of cells at various stages of differentiation. In uterine rhabdomyosarcoma, the expression of desmin, muscle-specific actin, myogenin, and MyoD1 is evident, and the expression status of these markers may be used as a reference for the diagnosis of uterine rhabdomyosarcoma [10].

Using immunohistochemical staining, strong expression of desmin, a molecular marker of muscle cells, was observed in the patient’s uterine tumor and uterine leiomyosarcoma. Desmin expression was not observed in normal uterine smooth muscle tissue (Figure 4). Strong expression of myogenin, a molecular marker of muscle cells, was observed in the uterine tumor; however, no expression of myogenin was observed in uterine leiomyosarcoma (Figure 4). The expression status of other epithelial cell and neuroendocrine markers was evaluated in the patient’s tumor. Based on these molecular pathological results, the uterine tumor was diagnosed as a pleomorphic rhabdomyosarcoma.

Rhabdomyosarcoma may be classified histopathologically into a fetal type, alveolar type, and pleomorphic type. Most rhabdomyosarcomas are fetal or alveolar, and occur most often in the head and neck, limbs, and genitourinary system of children. The pleomorphic type is believed to be more likely to occur in the limbs of the elderly [26]. For children, the International Rhabdomyosarcoma Study, which was established in the United States in 1972, has recommended combination therapy with surgery, chemotherapy, and radiation therapy [27].

According to recent data from the rhabdomyosarcoma study group in Europe, the United States, and Japan, the three-year progression-free survival rate is 80–100% in the low-risk group, 50–80% in the intermediate-risk group, and 30–50% in the high-risk group. Chemotherapy for fetal and alveolar rhabdomyosarcoma includes VAC therapy with vincristine (VCR), actinomycin D (ACD), and cyclophosphamide (CPA) as standard treatment. However, in adults, pleomorphic rhabdomyosarcoma often develops, and standard treatment has not been established. In particular, elderly people that do not respond to VAC therapy have a poor prognosis in many cases. Unfortunately, this case (Case 1: Supplementary Material S1) was a pleomorphic rhabdomyosarcoma and no response to VAC therapy was observed (Supplementary Material S1, Supplementary Material S3). In contrast, the case of embryonal rhabdomyosarcoma (Case 2: Supplementary Material S1) responded to VAC therapy (Supplementary Material S3).

Vimentin, cytokeratin, and ethylmalonic-adipicaciduria (EMA) are often used to distinguish epithelial tumors from non-epithelial soft tissue tumors. Vimentin is positive in many soft tissue tumors and negative in epithelial tumors. Cytokeratin and EMA are positive in epithelial tumors, but these markers tend to be positive in synovial sarcoma and epithelioid sarcoma, which have epithelial-like characteristics in soft tissue tumors. For pathological diagnosis, desmin, myoglobin, myogenin, MyoD1, α-smooth muscle actin, and HHF-35 are used as markers for myogenic tissues. The expression of desmin and HHF-35 is clearly observed in both striated and smooth muscle tumor tissue. Thus, the expression of desmin and HHF-35 is also observed in leiomyoma tissue, rhabdomyosarcoma tissue, benign leiomyoma, and nodular fasciitis tissue. Myogenin, MyoD1, and myoglobin are markers specific to striated muscle tissue. In particular, myogenin is useful for surgical pathological diagnosis because of its high sensitivity and specificity in rhabdomyosarcoma tissue, whereas α-smooth muscle actin is a marker specific for smooth muscle tissue.

Sarcomas are rare and usually display no specific line of differentiation, although rhabdomyosarcoma and leiomyosarcoma have been described [28,29]. Recent study shows that NT5DC2 is aberrantly upregulated in uterine leiomyosarcoma, and the expression of cyclin B1, cyclin A2, cyclin E1 is dependent on the expression of NT5DC2 [30]. However, the expression status of NT5DC2 in various mesenchymal tumors, including benign uterine leiomyoma, has not been clarified. Therefore, it is not clear whether NT5DC2 is a candidate biomarker for differentiating uterine leiomyosarcoma or rhabdomyosarcoma from other mesenchymal tumors. As pathogenesis for uterine leiomyosarcoma, the most frequently mutated genes include *TP53*, *ATRX*, and *MED12*; however no specific pathogenic variant has been identified [31,32]. On the other hand, molecular studies of a single case of pleomorphic rhabdomyosarcoma revealed *PIK3CA* and *TP53* mutations [33]. Therefore, it is inappropriate to distinguish between uterine leiomyosarcoma and uterine rhabdomyosarcoma based on the analysis results of pathological variants.

Previous research studies have indicated that the spontaneous onset of uterine leiomyosarcoma is observed in mice lacking LMP2/β1i, which is a subunit of the immunoproteasome [17,18,19]. In human uterine leiomyosarcoma, the expression of LMP2/β1i is significantly reduced [17,18,19]. However, as with normal uterine smooth muscle tissue and uterine leiomyoma, strong expression of LMP2/β1i was observed in the patient’s uterine rhabdomyosarcoma (Figure 1 and Figure 2, Table 1). Therefore, LMP2/β1i may be useful as a marker for differentiating uterine rhabdomyosarcoma from uterine leiomyosarcoma and other malignant mesenchymal tumors. The cytogenetic similarities detected thus far between leiomyoma and malignant muscle tumors (i.e., leiomyosarcoma and rhabdomyosarcoma) are few, which complicates the diagnosis of uterine leiomyoma and uterine rhabdomyosarcoma [34].

There are various theories regarding the origin of the development of uterine rhabdomyosarcoma [35], including Dr. Pfennenstiel’s theory (stromal cell metaplasia), Dr. Giorke and Nehrkorn’s theory (development of adult striated muscle in the bottom of the uterine), Dr. Wilms’ theory (development of lumbar mesenchymal cells along the Wolff canal), and the theory of Dr. Lahn and colleagues (origin of the Muller duct). Dr. Silverberg reported that rhabdomyosarcoma mixed with mesodermal components may develop from the mesenchymal tissue around the Muller duct [36]; however, the origin has not yet been determined [37].

From clinical studies to date, it is not uncommon to observe the development of malignant uterine leiomyosarcoma over the course of follow-up for benign tumor uterine leiomyoma. We experienced a case involving the development of uterine rhabdomyosarcoma from a benign tumor uterine leiomyoma. In this case, when making a surgical pathological diagnosis, the use of candidate biomarkers for uterine leiomyosarcoma assisted us in the differential diagnosis of a large uterine tumor. Currently, no clinical treatment for adult rhabdomyosarcoma has been established [38,39]. Further clinical studies should be conducted to improve the diagnosis and treatment of uterine rhabdomyosarcoma.

## 5. Conclusions

In uterine mesenchymal tumors, cells with various histological types and cell morphologies are mixed. Furthermore, the components contained within the cells also vary. Therefore, surgical pathological diagnosis for uterine mesenchymal tumor is not straight forward. Therefore, the information obtained by the molecular pathological diagnosis using putative biomarkers for uterine smooth muscle tumor is useful for the differential diagnosis of other uterine mesenchymal tumors. The case in the present study was diagnosed as uterine pleomorphic rhabdomyosarcoma by molecular pathological diagnosis using candidate biomarkers for uterine smooth muscle tumors.

## Figures and Tables

**Table 1:** Differential expressions of SMA, caveolin-1, cyclin B, cyclin E, LMP2, NT5DC2, CD133, and Ki-67 in human uterine mesenchymal tumors and uterine LANT-like tumor.

Mesenchymal Tumor Types	Age Years	n	Protein Expression *							
			SMA	CAV1	CCNB	CCNE	LMP2	NT5DC2	CD133	Ki-67
Normal	30–80 s	74	+++	-	-	-	+++	-	-	-
Leiomyoma (LMA) (Ordinally leiomyoma) (Cellular leiomyoma)	30–80 s	40 (30) (10)	+++	++	-/+	-/(+)	+++	-/+	-	+/-
			+++	++	-/+	-	+++	-/+		+/-
			++	++	-/+	-/(+)	++	-/+		+/-
STUMP	40–60 s	12	++	++	+	-/+	-/+	-/+	NA	+/+++
Bizarre Leiomyoma	40–50 s	4	++	++	-/+	+	Focal+	+	NA	+
Intravenous LMA	50 s	3	++	++	+	+	-	NA	++	+
Benign metastasizing	50 s	1	++	++	+	++	-	NA	NA	++
Leiomyosarcoma	30–80 s	54	-/+	+	++	+++	-/+	++	++	++/+++
Rhabdomyosarcoma	10 s, 50 s	2	NA	++	-/+	+++	+++	NA	NA	NA
U.LANT#-like tumor	40 s	1	++	+	NA	++	-	NA	NA	-

* Staining score of expression of SMA (smooth muscle actin), CAV1 (caveolin-1), CCNB (cyclin B), CCNE (cyclin E), LMP2 (low molecular protein 2), NT5DC2 (5’-nucleotidase domain containing 2) and Ki-67 from results of IHC experiments. Protein expression *; estimated-protein expressions by immunoblot analysis, immunohistochemistry (IHC) and/or RT-PCR (quantitative-PCR), -/+; partially positive (5% to 10% of cells stained), Focal+; Focal-positive (focal or sporadic staining with less than 5% of cells stained), ++; staining with 5% or more, less than 90% of cells stained, +++; diffuse-positive (homogeneous distribution with more than 90% of cells stained), -; negative (no stained cells). U.LANT-like tumor (uterine leiomyomatoid angiomatous neuroendocrine tumor-like tumor), LMP2 [13,20], cyclin E [13,20], caveolin-1 [13], NT5DC2 [21], CD133 [15], Ki-67 [13,20]. STUMP (smooth muscle tumor of uncertain malignant potential) [21,22]. Cyclin E, LMP2, caveolin-1 are potential biomarker for human uterine mesenchymal tumors. LANT ^#^, leiomyomatoid angiomatous neuroendocrin tumor (LANT) is described as a dimorphic neurosecretory tumor with a leiomyomatous vascular component [23]. NA; no answer.

## Data Availability

The data presented in this study are available on request from the corresponding author.

## Supplementary Materials

The following are available online at https://www.mdpi.com/article/10.3390/curroncol29040190/s1. Material S1: Case 1 and Case 2; Material S2: Results of surgical pathological diagnosis; Material S3: Primary Uterine rhabdomyosarcoma.

## Abbreviations

αSMA, alpha smooth muscle actin; CA125, ovarian carcinoma antigen-125; CD, clusters of differentiation; CT, computed tomography; DAPI, 40,6-diamidino-2-phenylindole; EMA, ethylmalonic-adipicaciduria; ER, estrogen receptor; IHC, immunohistochemistry; HHF-35, familial hyperinsulinemic hypoglycemia-35; LDH, lactic acid dehydrogenase; LMP2/β1i, large multifunctional peptidase 2/β1i; LMS, leiomyosarcoma; LYVE-1, lymphatic vessel endothelial hyaluronan receptor 1; MyoD1, myogenic differentiation antigen 1; PgR, progesterone receptor; WHO, World Health Organization.
