# Supplementary material for: Characteristic of Uterine Rhabdomyosarcoma by Algorithm of Potential Biomarkers for Uterine Mesenchymal Tumor
Source: Curr Oncol. 2022 Mar 28;29(4):2350–63. doi: 10.3390/curroncol29040190 (PMC9027675; doi:10.3390/curroncol29040190)

Supplementary Materials

Oncological features of intravenous leiomyomatosis: involvement of mesenchymal tumor stem-like cells

Saya Tamura, Takuma Hayashi, Tomoyuki Ichimura, Nobuo Yaegashi, Kaoru Abiko, Ikuo Konishi

Supplementary Material S1

Case 1: Patient background

In February 2003, Uterine Artery Embolization (UAE) was performed for a uterine leiomyoma in a 40-year-old woman at a nearby hospital. Because of subsequent excessive bleeding during the necrotic uterine leiomyoma delivery that developed under the mucosa, the vaginal leiomyoma was resected by short-term hospitalization or outpatient visit a total of 5 times from June 2003 to July 2009. During the histopathological diagnosis for resected uterine leiomyoma, it was determined that the cell density was rather high. No malignant findings were noted in the surgical pathological diagnosis and the patient was diagnosed with uterine leiomyoma with hemorrhage and necrosis. Afterwards, she did not feel any physical abnormalities with no complaints and did not go to the outpatient clinic for 12 years. At the age of 52, she had menopause. In March 2021, the abdominal bulge became evident and the blood clot was excreted from May 9 to 10, 2021. She received an outpatient visit at a nearby hospital. She was referred to our hospital because of a significantly larger mass in her abdomen. On May 31, 2021, she arrived at our hospital with a markedly swollen abdomen and swelling of the left supraclavicular lymph node.

Virchow: LNs: Palpate multiple bulky tumors

Abdomen: A neonatal-sized hard bulky tumor was found on the right side of the lower abdomen. This tumor has poor mobility

Vaginal discharge: small amount

Cervix: The cervix was not red and sore

Transvaginal ultrasonography (TV-USG): Only the area around the uterine ostium that is markedly swollen can be confirmed. The intensity of the echo is almost uniform. ascites (-)

Transabdominal ultrasonography (TA-USG): A bulky tumor measuring 246 x 140 mm in diameter, with a blood reservoir inside. Hemorrhagic necrosis and lumen are observed. An irregularly shaped ridge measuring 43 x 49 mm was found in the lumen.

CT and MRI images: The uterus is large enough to occupy most of the abdomen. Calcification due to some uterine leiomyoma is observed. Suspected onset of uterine leiomyosarcoma or uterine carcinosarcoma. Bulky tumors were found in the pelvis, para-aorta, and left supraclavicular lymph node.

Malignant lymphoma was suspected because of multiple lymphadenopathies.

Differential diagnosis: Pleomorphic rhabdomyosarcoma

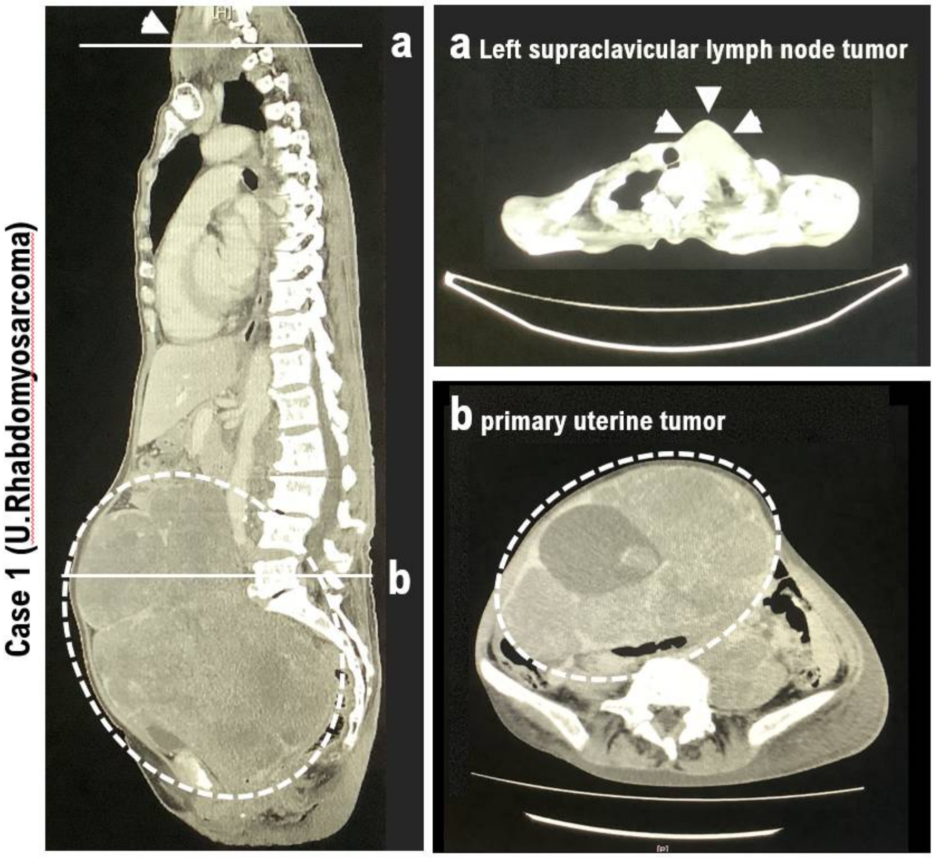

Contrast CT images
The mass of the left supraclavicular lymph node is indicated by the white arrowheads.
The white dotted circle indicates the uterine tumor.

Case 2: Patient background
A 16-year-old girl went to a general hospital for an outpatient visit because of lower abdominal pain and bleeding. Based on the results of various tests, she was suspected of having a malignant tumor originating from the uterus. The tumor was resected by a total abdominal hysterectomy and bilateral salpingo-oophorectomy. The surgical pathological diagnosis of the excised tissue indicated that the excised tumor was a uterine embryonal rhabdomyosarcoma. A response to VAC therapy was confirmed.

Supplementary Material S2
Case 1: Results of surgical pathological diagnosis
Diagnosis: Pleomorphic rhabdomyosarcoma
Tumor size: at least 23 cm in greatest dismension
Lymph-vascular invasion: present (LVI1)
Positive IHC: CD56 (partial), synaptophysin (partial), desmin (partial), myogenin (partial), AE1/AE3 (focal), Cam5.2 (focal), CD99 (partial, main dot-like), p53 (diffuse).
Negative IHC: EMA, chromogranin A.
Margins: uncertain
-Leiomyoma
Ovary and tube, bilateral, salpingo-oophorectomy: involved by pleomorphic rhabdomyosarcoma.

Omentum, biopsy: A small amount of necrotic cells and tumor cells are mixed.

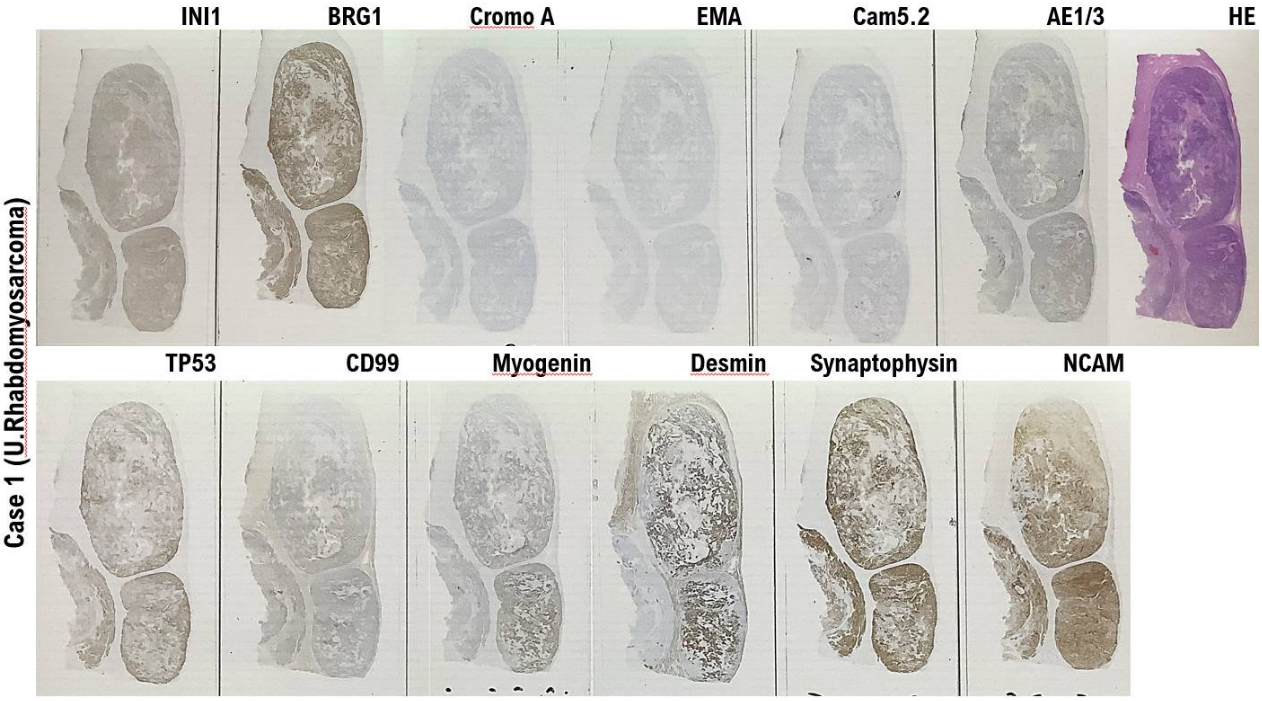

Supplementary Material S3

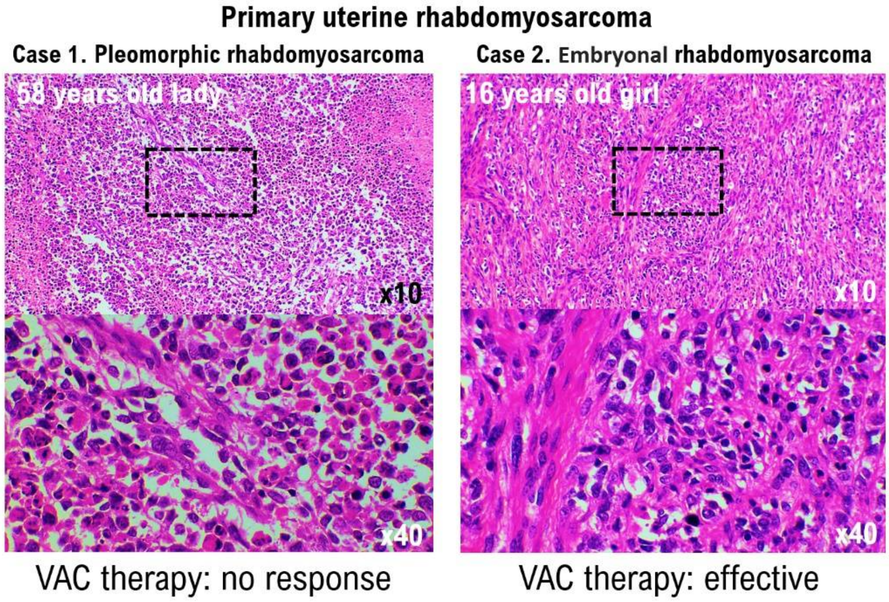

Supplement: Supplementary file 1 [file curroncol-29-00190-s001.zip › curroncol-1644828-supplementary.pdf]
